# Estimating Compositions and Nutritional Values of Seed Mixes Based on Vision Transformers

**DOI:** 10.34133/plantphenomics.0112

**Published:** 2023-11-10

**Authors:** Shamprikta Mehreen, Hervé Goëau, Pierre Bonnet, Sophie Chau, Julien Champ, Alexis Joly

**Affiliations:** ^1^ Inria, LIRMM, University Montpellier, CNRS, Montpellier, France.; ^2^ CIRAD, UMR AMAP, Montpellier, Occitanie, France.; ^3^ Chambre d’Agriculture - Haute Vienne, Limoges, Nouvelle-Aquitaine, France.

## Abstract

The cultivation of seed mixtures for local pastures is a traditional mixed cropping technique of cereals and legumes for producing, at a low production cost, a balanced animal feed in energy and protein in livestock systems. By considerably improving the autonomy and safety of agricultural systems, as well as reducing their impact on the environment, it is a type of crop that responds favorably to both the evolution of the European regulations on the use of phytosanitary products and the expectations of consumers who wish to increase their consumption of organic products. However, farmers find it difficult to adopt it because cereals and legumes do not ripen synchronously and the harvested seeds are heterogeneous, making it more difficult to assess their nutritional value. Many efforts therefore remain to be made to acquire and aggregate technical and economical references to evaluate to what extent the cultivation of seed mixtures could positively contribute to securing and reducing the costs of herd feeding. The work presented in this paper proposes new Artificial Intelligence techniques that could be transferred to an online or smartphone application to automatically estimate the nutritional value of harvested seed mixes to help farmers better manage the yield and thus engage them to promote and contribute to a better knowledge of this type of cultivation. For this purpose, an original open image dataset has been built containing 4,749 images of seed mixes, covering 11 seed varieties, with which 2 types of recent deep learning models have been trained. The results highlight the potential of this method and show that the best-performing model is a recent state-of-the-art vision transformer pre-trained with self-supervision (Bidirectional Encoder representation from Image Transformer). It allows an estimation of the nutritional value of seed mixtures with a coefficient of determination *R*^2^ score of 0.91, which demonstrates the interest of this type of approach, for its possible use on a large scale.

## Introduction

The cultivation of seed mixtures for local pastures is a promising approach to produce a balanced animal feed in energy and protein for both conventional and organic livestock systems [1,2]. This technique involves planting seed mixtures as a winter crop between 2 main crops, offering advantages such as limiting biotic pressures, reducing the risk of crop failure, controlling pests, and enhancing soil fertility. However, the adoption of this cultivation method has been challenging for farmers due to the heterogeneity of harvested seeds and the difficulty in assessing and controlling their nutritional potential. Moreover, the simultaneous maturation of cereals and legumes for appropriate harvesting presents further complexity.

Current practices of mixed cropping cereals and legumes have been based on intuitive and empirical methods, as well as advice between farmers. To promote this technique and maximize its benefits, efforts are needed to aggregate technical and economic references for reducing pesticide use and estimating the impact on the herd’s diet in terms of volumes and nutritional values, along with identifying potential resource savings.

Thus, a primary scheme is to engage a substantial number of farmers and experts in the field to collect raw data and experiences about their practices. These data will be used to promote mixed cropping as an efficient technique and address challenges related to seed mixtures. Inspired by the success of Pl@ntNet, a participatory citizen science platform for collecting, sharing, and reviewing plant observations based on automated identification [3], it is assumed that a mobile application or online service capable of automatically estimating the nutritional value of harvested seed mixtures from a photo could be a key element in promoting this type of agriculture. The more accurate the estimation algorithm becomes, the more farmers will be willing to use this service, as it will help them to become more proficient in managing the yield of their fields. Once convinced of its benefits, they will be more willing to participate in enriching a common database of annotated photos, providing information on seed proportions and varieties. As this database becomes richer and more diverse in terms of proportions, seed varieties, camera qualities, shooting conditions, and agricultural sites, the algorithm will further improve and attract new farmers to utilize the application.

The pursuit of this scheme necessitated careful consideration and resolution of a wide range of diverse challenges, both in the agriculture and in the computer vision domains. The primary challenge involved successfully gathering a sufficient number of farmers who were convinced of the approach and willing to coordinate, cultivate, collect, and weigh quantities of specific seed varieties on a portion of their agricultural land over several years. Subsequently, the issue of capturing images of the cultivated seeds arose. After briefly discarding the idea of direct on-field photography, the challenge remained of finding a method to capture images of seed mixtures that would not require the purchase of specialized equipment or impose excessive shooting efforts. Then, concerning the construction of training and validation sets to compare different methods, the challenge was to ensure that the evaluations were credible regarding the ability of an estimation system to generalize in terms of new data and new seed mixture compositions and proportions that have not yet been encountered in the training set. It was also essential to consider a common pitfall in creating evaluation biases, where images with very similar compositions, such as those from the same cultivated field, could inadvertently end up scattered between the training set and the validation set, leading to overly optimistic evaluations regarding the true generalization capability and performance of an estimation system.

Finally, the candidate methods at the core of the nutritional value estimation system encounter various challenges in image analysis techniques. Approaches like semantic segmentation or instance segmentation [4–7] have shown efficacy in agronomy tasks requiring object counting for food production control [8–10]. However, the manual annotation for each object in the training set hinders these methods’ use in cases with cluttered scenes and numerous indistinguishable objects, as seen in this seed proportion estimation work. The seeds’ abundance, overlapping, and changing appearance due to orientation make manual annotation time-consuming. To avoid this, a global approach was adopted, employing deep neural networks typically used for image classification, repurposed for a regression problem. The goal is to directly predict the weight percentages of different seed varieties in a mix. This approach only requires information about the mix composition for training, whereas segmentation methods provide composition estimates based on coverage in the image. This enables us to achieve more relevant results and enhance the method’s transferability to different contexts, including other crops. However, there are many competitive models in image classification, based on convolutional neural networks (CNNs) [11] or vision transformers (ViTs) [12], and it is difficult to state in advance which type of architecture is the most relevant in this particular context of a regression problem based on the analysis of small objects. In addition, it is important to consider what loss function should be retained when training a model beyond the usual cross-entropy used for image classification, to ensure, for example, that a model is capable of predicting a probability of zero in the absence of a variety. Hence, we experimented with different loss functions to get the desired predictions.

To accomplish this ambitious long-term project, this paper introduces the foundational milestones to initiate a sustainable virtuous cycle. The objectives of the work presented in this paper are as follows: (a) collaborate with a group of willing farmers to cultivate, collect, and weigh quantities of seed varieties; (b) define a protocol for acquiring seed mix images; (c) build a comprehensive training and validation dataset; (d) identify and adapt state-of-the-art methods for evaluating and comparing performance with relevant evaluation metrics; and (e) implement the most effective approach into a real, online production system accessible to everyone.

## Materials and Methods

### Field data acquisition

**Fig. 1.**
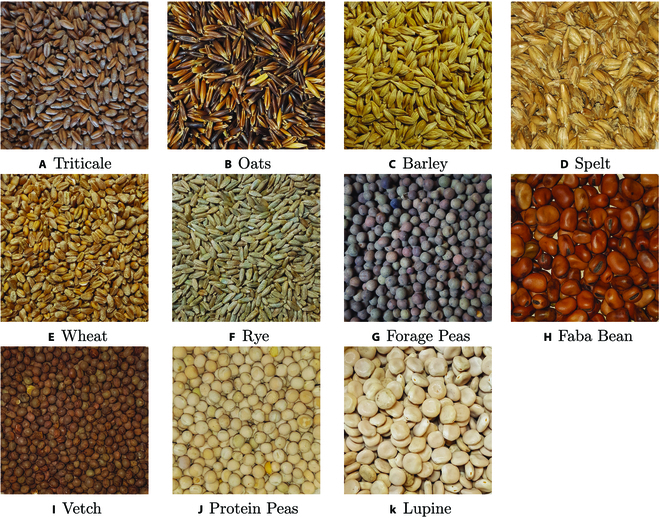
The 11 cereal and legume cultivated varieties of seeds. (A) Triticale. (B) Oats. (C) Barley. (D) Spelt. (E) Wheat. (F) Rye. (G) Forage peas. (H) Faba bean. (I) Vetch. (J) Protein peas. (K) Lupine.

The work was carried out as part of the Carpeso project [13], which collaborates with several French agricultural institutes to establish a shared database containing the results of characterizing the feed value of organic and conventional mixed crops involving cereals and legumes. Over a 3-year period, from 2019 to 2022, an extensive crop harvesting and photo collection campaign was carried out. This campaign involved a network of over 20 farmers located in the southwestern quarter of France, resulting in the acquisition of over 4,000 images. These images showed a wide variety of proportions relative to 11 cereal and legume varieties frequently used for animal feed in this region of France (Fig. 1).

During the first year of the Carpeso project, the farmers were asked to directly cultivate “Real mixes”. They sowed mixtures of cereals and/or legumes in their fields and harvested and cleaned the seeds once they reached maturity. However, after this preparation process, there was often residual debris, such as small pieces of stems, seed husks, or unidentified seeds, which collectively represented a considerable mass. These remnants were weighed and categorized as a 12th category called “other” in addition to the 11 main varieties.

**Fig. 2.**
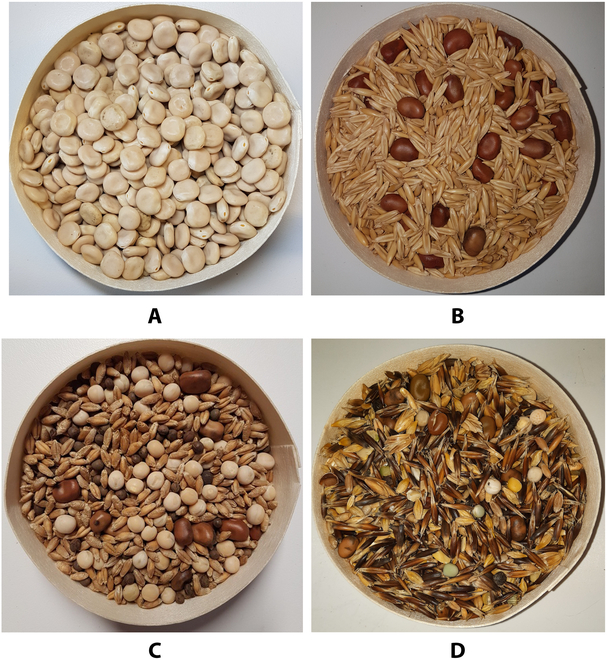
Four kinds of collected images containing different seed mixtures. (A) Monocrop (100% lupine). (B) Composition of seeds of two monocrops (75% oats and 25% faba bean). (C) Composition of seeds of 4 monocrops (60% triticale, 20% forage peas, 10% faba bean, and 10% vetch). (D) Real Mix (42% oats, 14% triticale, 14% faba bean, 11% barley, 7% spelt, 6% forage peas, and 6% others).

Subsequently, in collaboration with the farmers, a relatively simple image capture protocol was defined using a standard french camembert cheese box (Fig. 2). The farmers were instructed to first take a sample of a seed mixture and photograph the box at the center of the image against a uniform background (such as a table, paper, or fabric). Seed weighing was performed using standard GBK16 balances. Each mix was sampled 5 times, and each sample was photographed 5 times by shaking the box between each photo to enhance the visual diversity.

After this initial data collection in 2019, a total of 465 images (Table 1) were photographed and weighed. Manual weighing was a time-consuming and labor-intensive process, taking approximately 2 h per mix type, noticeably slowing down data production.

Data collection for real mixes continued over the following 2 years, but to expedite data production, a 2-year plan was devised in collaboration with the farmers. In 2020, the farmers first cultivated monocrop fields, each containing a single type of variety. The following year, the monocrop harvests from the previous year were intentionally mixed in a controlled manner. This approach gradually introduced mixtures with proportions that did not exist in the previous years.

**Table 1.** Image acquisition throughout the years 2019, 2020, and 2021.

Year	Monocrop	Composition of monocrops	Real mixes	Total
2019	0	0	465	465
2020	1,228	0	659	1,887
2021	0	2,243	154	2,397
Total	1,228	2,243	1,278	4,749

### Data analysis

**Fig. 3.**
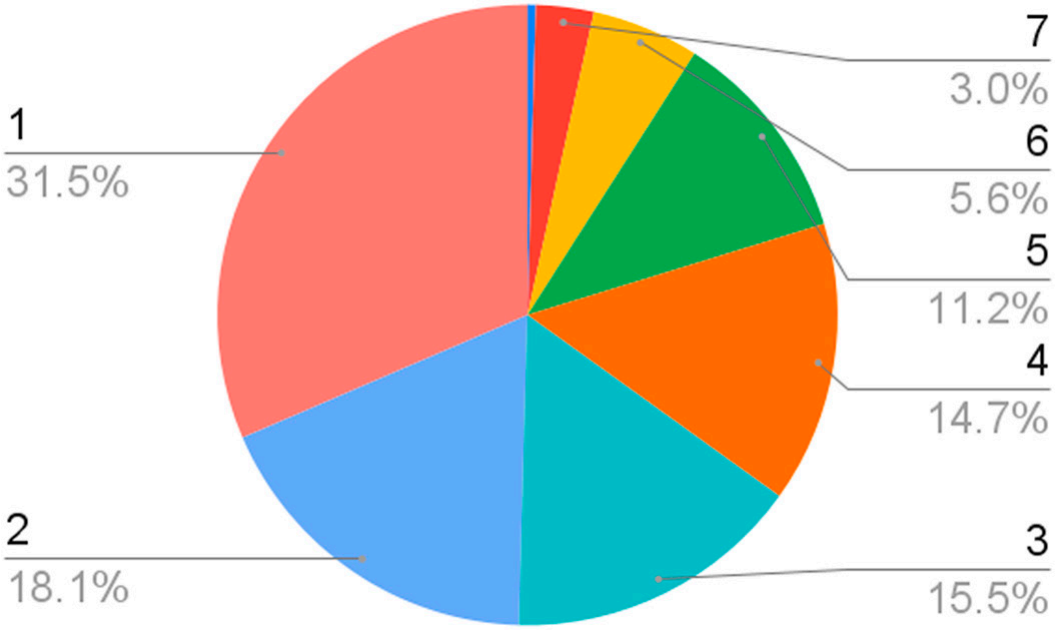
Distribution of the number of seed varieties in the mixes (18.1% of the mixes contain 2 types of seed varieties).

**Fig. 4.**
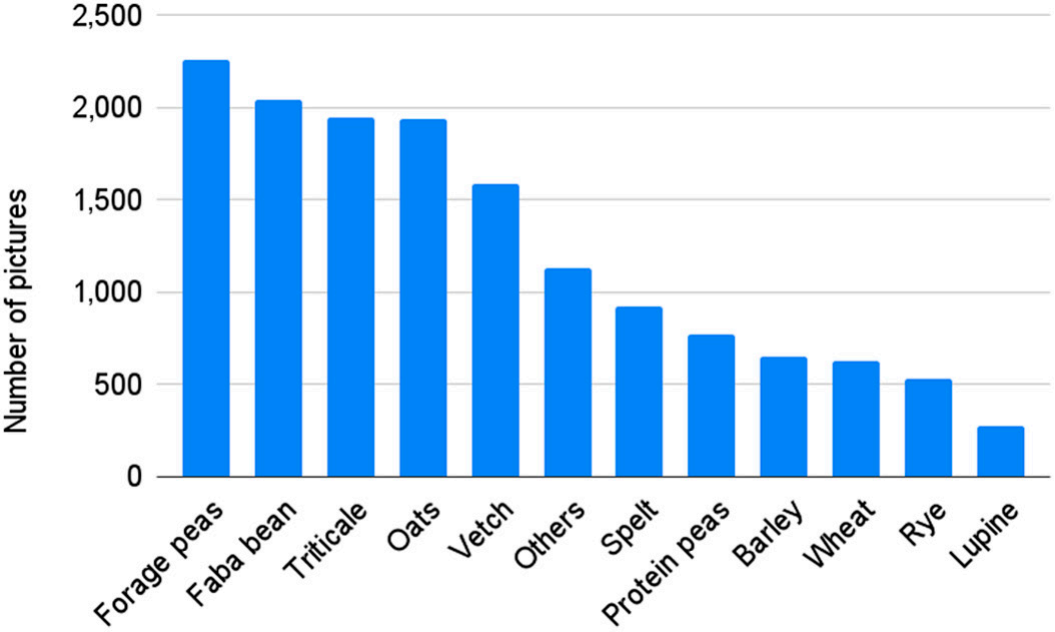
Number of pictures per variety presence.

In the end, a total number of 4,749 images were collected from 205 seed mixes, including the ones based on the composition of monocrops and the real ones harvested from intercropping fields. The samples contain from 1 to 7 types of seed varieties (Fig. 3), but the majority contains less than 5 types. There are a total of 11 different types of varieties of cereal and legume: triticale, oats, barley, spelt, wheat, rye, forage peas, protein peas, faba bean, vetch, and lupine. Forage pea is the most frequent variety while lupine is the least frequent (Fig. 4). The additional class “others” is for any item other than the previously mentioned 11 types of seed categories, such as residual weed or crop fragments.

Finally, beyond the aspects of proportion and type of mixed varieties, the collected data encompass a diverse range of acquisition conditions, including the use of different cameras with different qualities and images taken at different times of day with different lighting conditions, both indoors and outdoors.

### Deep learning models dedicated to seed weight estimation

Instead of relying on segmentation approaches, the chosen approach involves direct prediction of the composition from the image. One of the key advantages of this method is that it solely relies on information about the composition of the mixes for model training. Specifically, the weight composition can be used directly as the learning objective, whereas segmentation approaches can only estimate composition in terms of coverage within the image. The approach involves adapting deep neural networks, originally designed for image classification, to a regression task. The primary objective is to directly estimate the weight proportion of each crop within the mix using the entire visual content of the image. The hypothesis is that recent deep neural network architectures, including ViTs [14], can effectively train models that capture sufficient global and local information about the structure and appearance of the image. This enables the extraction of relevant features and the inference of proportions for different seeds. It is important to note that once the weight composition is estimated, the nutritional value of the mixture can be easily derived from the known nutritional values of each individual crop variety. Two main types of deep learning models used in computer vision are compared in this study, CNNs and ViTs.

#### Convolutional neural networks

In the last decade, CNNs [11] have been widely used in computer vision tasks. They have been consistently showing groundbreaking results in various machine learning problems including pattern recognition, image classification, image and video recognition, image segmentation, medical image analysis, etc. In this work, to have a point of comparison with the use of ViTs, one of the most competitive CNN architecture has been selected in terms of performance, computing time, and memory footprint belonging to the EfficientNet family of networks [15]. EfficientNet networks are based on the automatic search for optimal architectures by varying the input resolution of the network, the width, and depth of the different layers. This search resulted in a set of 7 models of different dimensions, which exceeded the state-of-the-art accuracy of most CNNs with much higher efficiency. More precisely, the fourth model called EfficientNet-B4 but pretrained with a “noisy student” approach [16] is chosen that refers to a semi-supervised training method, notably improving the accuracy of any EfficientNet model. Thus, such a model pre-trained on the ImageNet dataset represents a better starting point for further refinement on a more specific task as addressed in this study.

#### Vision transformers

ViTs are inspired by the popular Transformer approach in the field of natural language processing [17]. Transformers are designed to solve word sequence processing tasks capable of handling long-distance dependencies through self-attention mechanisms that preserve word interdependence in the sequence representation [17]. For computer vision, after several propositions of hybrid architectures combining self-attention mechanisms with convolutional operations, a pure Transformer approach was finally introduced in Ref. [12]. This first version of ViT directly applies a transformer to sequences of image patches and achieves equivalent results on various image classification datasets compared to state-of-the-art CNNs while requiring substantially fewer computational resources to train. In a ViT, an image is split into fixed-size patches, each of them is then linearly embedded, position embedding is added, and the resulting sequence of vectors is fed to a standard Transformer encoder. An interesting property of ViTs is that they relax the translation invariance constraint and the locally restricted receptive field of CNNs, meaning that ViT is more suitable to capture the global organization of the objects in the image [14].

The superiority of ViTs over CNNs in terms of performance has since been observed thanks to the introduction of new self-supervised learning (SSL) methods particularly suitable to ViTs. In the context of computer vision, the general idea behind an SSL technique is to force a network to predict any hidden part of an image from an unhidden part. The hidden part can rely on zooming and cropping such as in DINO (self-**di**stillation with **no** labels) [18], or rely on partial masking of image patches such as in Masked AutoEncoders (MAE) [19] or the Bidirectional Encoder representation from Image Transformer (BeiT) SSL method [20] that was selected and evaluated in this study. Once a model is pretrained with the SSL method (on a huge amount of unlabeled images), it can be then more efficiently fine-tuned in a classical supervised way on datasets with image labels (such as ImageNet). This procedure results in a new, better pre-trained model that can subsequently be refined for various downstream tasks, just as demonstrated in this study.

### Estimation of nutritional values

Several units of nutritional value are usually considered by agronomists and farmers. Among the most common is the starch content in grams per kilogram of dry matter (considering all constituents of matter except water). For this study, the unit milk fodder unit per kilogram of dry matter (MFU/kgDM) has been focused on, which is the amount of net energy that can be absorbed during lactation or maintenance of a ruminant (1 MFU corresponds to 1,700 kcal). Once a model is trained, the nutritional values can be estimated by a weighted combination of the predicted proportions of seeds given by the model with the individual nutritional values of the seed varieties provided by the Carpeso consortium shown in Table 2.

**Table 2.** Nutritional values of the 11 seed varieties expressed as the milk fodder unit per kilogram of dry matter.

Seed variety	MFU/kgDM
Barley	1.09
Faba bean	1.26
Forage peas	1.25
Lupine	1.34
Oat	0.99
Protein peas	1.25
Rye	1.19
Spelt	0.93
Triticale	1.17
Vetch	1.26
Wheat	1.19

While calculating the nutritional values for the seed mixtures, the estimations have been made both on the image level and on the mix level, i.e., calculating the nutritional values directly from the images containing different seed compositions (image level) and calculating the nutritional values from seed mixes while each mix contains a different number of images, i.e., pictures from the same bag of harvested seed (mix level). In the “Nutritional values estimation” section, Table 9 presents different evaluations on calculating the nutritional values over the validation set both on the image level and mix level, and Fig. 8 presents the comparison between the nutritional value predictions on image level and mix level.

### Loss functions

The task that is desired to optimize is the prediction of the weight composition of a given input mix. The training data are composed of a set of pairs (**x**, **y**), **x** representing the input image of the mix and **y** ∈ *ℝ^d^* representing the target, i.e., the vector of the relative weights of each of the *d* = 11 considered varieties. Since **y** can be interpreted as a categorical distribution, the first solution to measure the error between the prediction $\hat{y}$ and the target **y** is to use the Kullback–Leibler (KL) divergence [21]. It is indeed suitable for minimizing the dissimilarity between any 2 (probability) distributions in particular categorical distributions. However, the KLDiv loss used on top of a classical softmax function is not suitable to infer sparsity, i.e., to ensure null predictions when several categories have to be predicted with perfect zero probability like in the application. Thus, in this study, an alternative loss function based on sparsemax [22] has also been studied. The sparsemax loss function is a variant of the softmax function used in machine learning, particularly in the context of multi-class classification tasks. The key characteristic of sparsemax is that it tends to assign zero probability to a large part of the classes, effectively “sparsifying” the output (as expected since the majority of the mixes have less than 4 seed varieties present in them).

### Training and validation sets, data augmentation, and evaluation metric

The collected dataset contains 205 seed mixtures, and each mixture was photographed 25 times on average, for a total of 4,749 images. For training CNN and ViT models, the seed mixtures were split randomly into a training and a validation set with a ratio of 80%–20%. This prevents images of the same mixture from being found in both the training set and the validation set, thus reducing the risk of bias in the evaluation (Table 3).

**Table 3.** Training and validation sets.

Dataset	Seed mixtures	Images
Training set	163	3,805
Validation set	42	944
Total	205	4,749

**Fig. 5.**
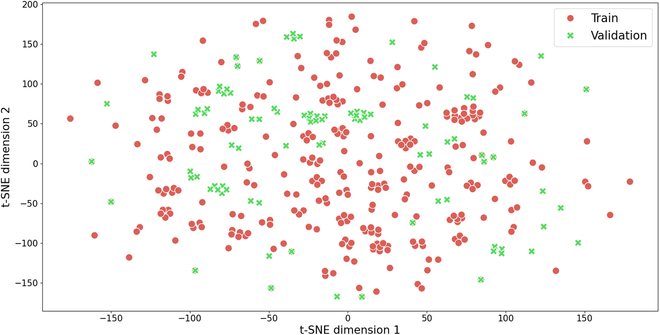
2D t-SNE projection of train and validation data distribution.

Figure 5 provides a 2D projection of data distribution in both the training and validation sets, and it demonstrates a noteworthy diversity in the compositions of seed mixtures within the training set. Additionally, it is evident that a considerable portion of the compositions present in the validation set are not adequately represented in the training set.

To limit the risk of over-fitting the models and improve their robustness, several data augmentation techniques were used including center crop, random rotation, color jitter, and auto contrast, as well as RandAugment [23] and AutoAugment [24], 2 distinct automated data augmentation methods designed to improve the generalization of deep learning models, especially suitable for small training datasets.

For evaluating the models, the Mean Absolute Error (MAE) was used as the primary metric, which is basically the average of the absolute value of the difference between the predicted seed proportions and the real seed proportions in the ground truth. As complementary metrics, the Mean Squared Error (MSE) was used, which penalizes the largest prediction errors more. Additionally, the *R*^2^ score is reported, which is classically used in regression problems. The *R*^2^ score evaluates the quality of the predictions by calculating the ratio between the total error of a trained model and the total error of a poorly performing model that would always predict the same average prediction values. Another complementary metric that was used for the evaluation is the max MAE/seed mentioned in Results, which focuses on the seed category with the highest MAE, giving us some insight into which individual seed category performs the worst and influences the global MAE.

### Training procedure and hyperparameters

For the experiments with the BeiT-base-384 model, the chosen optimizer was the SGD (Stochastic Gradient Descent) with a scheduler Reduce on Plateau (patience = 5), and a learning rate of 0.00005 for a batch size of 10. Considering the size and number of trainable parameters, a similar learning rate and batch size ratio was maintained for the the other models. Using a standard workstation of Nvidia Titan RTX, it took 6 h to train each EfficientNet-B4-ns model, 3 ^1^/_2_ h to train each BeiT-base-384 model, and 26 h to train each BeiT-large-512 model.

## Results

### Overall performance

Table 4 reports the 4 metrics comparing the 2 types of architectures CNN and ViT retained for the study. The results show a clear difference in performance in favor of the ViT-based architecture with a mean absolute error of only 0.0383 for the BeiT-base-384, an error reduced by ∼3% compared to the CNN EfficientNet-B4-ns. The other 3 metrics are even more favorable to ViT, demonstrating lower MSE, MAE for the seed variety (vetch) with the maximum error, and a much higher *R*^2^ score. It should be noted that even if the architectures of the 2 models are deeply different, they are comparable in terms of input image size (380 × 380 pixels for EfficientNet-B4-ns and 384 × 384 for the BeiT-base-384).

**Table 4.** Results on the validation set comparing EfficientNet and ViT-based models using KLDiv loss during the training (best score in bold).

Model	MAE	MSE	*R*^2^ score	Max MAE/seed
EfficientNet-B4-ns	0.0671	0.0184	0.3748	0.3157 (Vetch)
BeiT-base-384	**0.0383**	**0.0077**	**0.6909**	**0.3039 (Vetch)**

Since the BeiT model outperformed the EfficientNet model for predicting the proportions of the seeds in the seed mixtures, the next experiments were focused on the ViT architecture considering the BeiT-base-384 result as the baseline and trying to make the most of the performance. Table 5 represents the impact of the 2 data augmentation techniques RandAugment [23] using the configuration “rand-m9-mstd0.5” and AutoAugment [24] using the policy V0. Both techniques improved the performance, with at best a reduced MAE of 0.037 using AutoAugment.

**Table 5.** Results on the validation set using different data augmentations using KLDiv loss during the training (best score in bold).

Model	MAE	MSE	*R*^2^ score	Max MAE/seed
BeiT-base-384 without Augmentation	0.0383	0.0077	0.6988	0.3039 (Vetch)
BeiT-base-384 + RandAugment	0.0375	0.0075	0.7100	0.3056 (Vetch)
BeiT-base-384 + AutoAugment	**0.0370**	**0.0068**	**0.7242**	**0.3015 (Vetch)**

Afterward, the extent to which a larger model size could impact the results was assessed. The BeiT base model (86M parameters, 384 × 384 as image input size) was compared with the large version (307M parameters, 512 × 512 as input image size). Table 6 shows the improvement in the MAE when using the large version of BeiT, but not in terms of MSE and *R*^2^ score. However, training the BeiT-large was time-consuming compared to the other models, nearly 8 times slower than the BeiT base version. Considering the metric scores and the training time, the base version of BeiT was chosen to use for the rest of the experiment.

**Table 6.** Results on the validation set comparing 2 different sizes of the BeiT architectures using KLDiv loss during the training (best score in bold).

Model	MAE	MSE	*R*^2^ score	Max MAE/seed
BeiT-base-384 + AutoAugment	0.0370	**0.0068**	**0.7242**	0.3015 (Vetch)
BeiT-large-512 + AutoAugment	**0.0348**	0.0071	0.7213	**0.2854 (Vetch)**

The last component of the model that was evaluated was the choice of the loss function. Table 7 presents the results achieved with respectively the classical KLDiv loss (computed on top of the softmax) and the Sparsemax variant aimed at forcing the sparsity of the predicted probabilities. While the global MAE did not seem to improve using the Sparsemax loss, the individual MAE for the seed variety vetch seems to improve by 4%. The reason behind such a result is that while the Sparsemax loss can enforce zero probabilities focusing only on the relevant classes, it dismisses the seed varieties with relatively minor representation in an image. On the other hand, Softmax can represent mixed predictions by assigning relatively lower probabilities to less confident varieties. Although the images never contain all the seed varieties, there are a large number of zeros present in the dataset (∼56%), and there are also a large number of compositions with lower quantities of seed varieties. Thus, the overall MAE using softmax outperforms sparsemax, whereas the MAE for individual seed varieties seems to perform slightly better with Sparsemax, considering the improvement of seed variety vetch on the max MAE per seed. Nevertheless, considering that Softmax outperformed Sparsemax slightly in terms of the global MAE and the fact that BeiT-base-384 is considerably faster to train than BeiT-large-512, the model BeiT-base-384 + AutoAug + KLDiv was deemed the best-performing model for this study.

**Table 7.** Results on the validation set comparing the KLDiv and the Sparsemax loss (best score in bold).

Model	MAE	MSE	*R*^2^ score	Max MAE/seed
BeiT-base-384 + AutoAug + KLDiv	0.0370	**0.0068**	**0.7242**	0.3015 (Vetch)
BeiT-base-384 + AutoAug + Sparsemax	0.0387	0.0077	0.7144	**0.2625 (Vetch)**
BeiT-large-512 + AutoAug + KLDiv	**0.0348**	0.0071	0.7213	0.2854 (Vetch)

### Detailed results by seed varieties

**Fig. 6.**
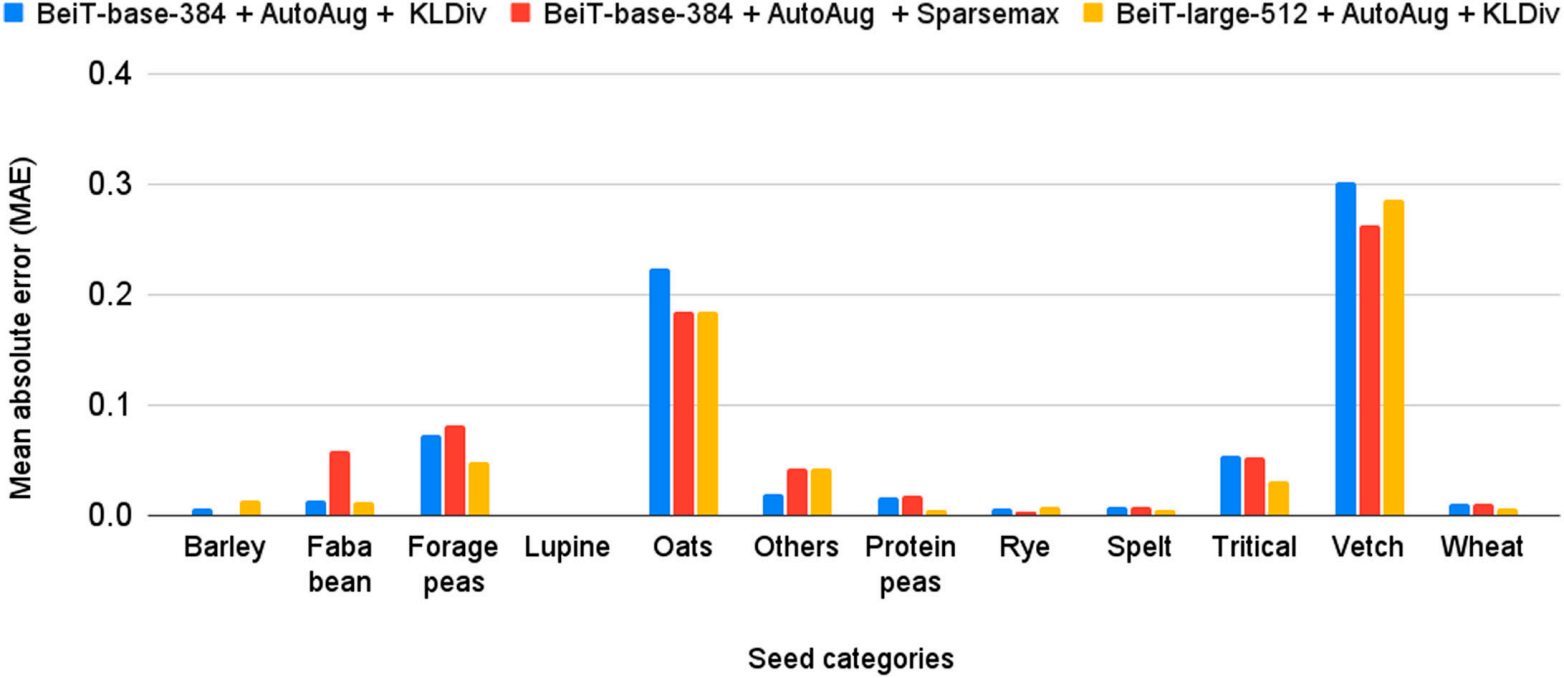
Comparison of MAE for each seed category using different model architectures and loss functions.

**Fig. 7.**
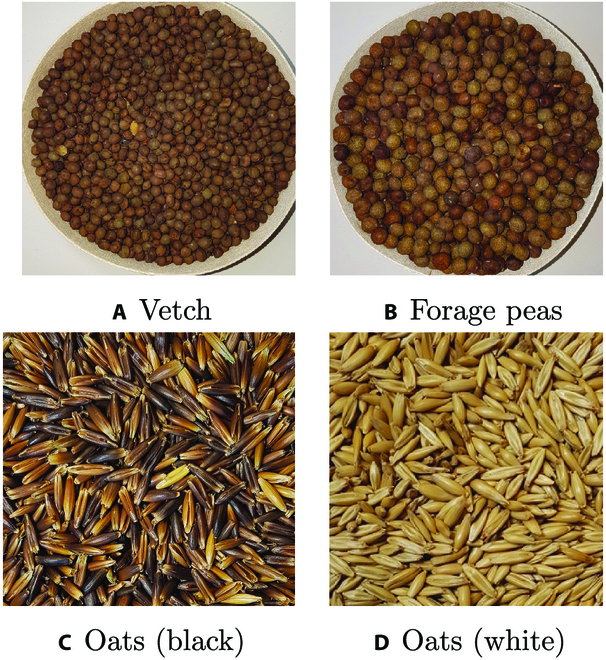
Illustrations related to categories with highest errors: (A and B) visual similarities between 2 distinct seed categories (vetch and forage peas); (C and D) visual diversity within the oat category.

Figure 6 presents the MAE values per individual seed varieties for the 3 models mentioned in Table 7. It shows that the performances of the 3 models are significantly different for each seed variety. In particular, every model performs very well for the seed categories like barley, lupine, rye, spelt, and wheat whereas they have more difficulties predicting vetch and oats. for vetch, one possible reason is that the models are more likely to confuse them with forage peas because they are visually quite similar and difficult to distinguish even for human eyes as illustrated in Fig. 7. The same possible reasoning can be established for the seed variety triticale, which is a hybrid of wheat and rye and is visually very similar to wheat. For the category oats, one explanation might be the co-existence of 2 quite visually distinct sub-categories (“black oats” or “white oats”). In particular, the “white oats” can potentially be confused with barley (Fig. 1).

### Results on mix-level predictions

The mix-level prediction is calculated by taking an average of multiple predictions of the same observation. The main objective of calculating the predictions on the mix level is to make the predictions more robust. Performances seem to be significantly improved while aggregating by averaging the predictions of all images related to each mix, i.e., containing a series of around 25 images related to the same mix. Table 8 gives the comparison between the results obtained by calculating different errors on the validation set for the predictions on image level and mix level. Here, the given predictions are from the model BeiT-base-384 + AutoAug + KLDiv, since it seems to provide a balance between the performance and size (Table 7). On the mix-level predictions, in addition to the global MAE and other metrics, the individual MAEs per seed seem to decrease by ∼21%

**Table 8.** Results on the validation set comparing predictions on Image level and Mix level using BeiT-base-384 + AutoAug + KLDiv (best score in bold).

	MAE	MSE	*R*^2^ score	Max MAE/seed
Image-level predictions	0.0370	0.0068	0.7242	0.3015 (Vetch)
Mix-level predictions	**0.0348**	**0.0055**	**0.7808**	**0.0898 (Triticale)**

### Nutritional values estimation

**Fig. 8.**
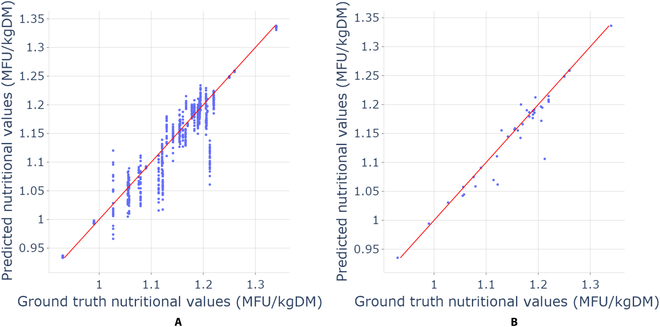
Nutrition value estimation on image level (A) and nutrition value estimation on mix level (B).

Figure 8 represents the true nutritional values and predicted nutritional values on (Fig. 8A) image level and (Fig. 8B) mix level present in the validation dataset. From the plot, it can be observed that the model tends to slightly underestimate the nutritional values of some samples but that it is quite accurate for the majority of samples. To evaluate the predicted nutritional values produced by the chosen model BeiT-base-384 + AutoAug + KLDiv, similar metrics were used to measure the performance of the proportions of seed mixtures 3.1. Table 9 shows the improvement of performance on the mix-level predictions and an *R*^2^ score of 91%.

**Table 9.** Results on the validation set comparing nutritional value predictions on image level and mix level (best score in bold).

	MAE	MSE	*R*^2^ score
Image-level predictions	0.0170	0.0007	0.8768
Mix-level predictions	**0.0131**	**0.0005**	**0.9103**

From the result, it can be concluded that ViTs pre-trained in a self-supervised way (BeiT), are significantly more accurate compared to CNNs. The most likely reason for this improved performance is that ViTs are able to capture at the same time globally and locally structured content facilitating the detection and counting of the individual seeds. Augmentation techniques slightly improved the performances. Larger network architectures, in terms of image resolution and number of parameters, brought a few improvements but at the cost of too much higher computing resources.

In addition to the usual KLDiv loss for regression problems, the Sparsemax loss was experimented with, assuming that it would help a model to better learn and predict the majority of cases where many seed varieties are absent from the mixes (almost 80% of the mixes have less than 4 seed varieties, which is representative of real scenario). However, despite the sparsity of the dataset, better performances were still achieved with the classical KLDiv loss than the Sparsemax loss. According to the experiments, aggregating the predictions of several pictures coming from the same mix (i.e., same bag of harvested seeds) is a good strategy to make the predictions more robust and less sensitive to seed varieties that are difficult to recognize such as vetch or oat. Finally, when estimating the nutritional value by converting the predicted proportions, an *R*^2^ score of 91% was achieved. According to the experts from the Carpeso project, this score is already largely acceptable.

A web component called “ESTI’METEIL” was developed with open access, enabling experimentation with the best model through a user-friendly interface (https://c4c.inria.fr/carpeso/). This component allows users to submit 1 to 5 images of a specific seed mix and receive the corresponding estimated seed composition (by weight) and nutritional value. The use of responsive design technology ensures that the web component is easily accessible and usable on mobile devices, making it convenient for farmers to test and utilize.

## Conclusion

This study explores the application of state-of-the-art deep learning techniques to address the challenge of estimating the composition and nutritional value of seed mixtures used in local pastures. The Carpeso project aimed to enhance the adoption of mixed cropping techniques, which offer cost-effective and environment-friendly solutions for livestock feed production in compliance with evolving European regulations and consumer demands for organic products. By creating an original open image dataset, incorporating a diverse range of seed varieties, and training state-of-the-art deep learning models, particularly the self-supervised ViT (BeiT), this study demonstrates promising results.

The effectiveness of the BeiT model in accurately estimating the nutritional value of seed mixtures is clearly distinguishable. ViTs, with their capacity to simultaneously capture global and local structured content, are excellent in detecting and counting the individual seeds, enabling them a superior choice compared to traditional CNNs. Furthermore, the application of augmentation techniques shows a slight performance improvement, while larger network architectures, though offering some benefits, come at the cost of higher computational resources. To address the sparsity of the dataset and the prevalence of mixes with a limited number of seed varieties, different loss functions, such as Sparsemax, were explored. Surprisingly, the classical KLDiv loss proved to be more effective in this context, leading to better model performance. Moreover, the proposed approach’s robustness was enhanced by aggregating predictions from multiple images taken from the same seed mix, mitigating challenges in detecting certain seed varieties, such as vetch or oat. As a result, an impressive coefficient of determination (*R*^2^) score of 0.91 was achieved when estimating the nutritional value, signifying the potential of the method for widespread use in practical applications.

The research presented in this paper introduces innovative techniques that have the potential to revolutionize the way farmers manage seed mixtures. The achieved results in this work are already widely considered satisfactory and the online prototype is ready to be used. Future work will focus on improving the balance of training data to ensure comprehensive coverage of various potential compositions. Additionally, another research perspective involves exploring the synthesis of training images to fill data gaps without requiring new data production in the field. This approach has the potential to enhance the model’s performance by augmenting the training dataset effectively.

## Data Availability

The images and the metadata used for the experiments are available in a package on Zenodo at https://zenodo.org/record/8169473 (DOI 10.5281/zenodo.8169473 accessed in July 2023).
