# Supplementary material for: Estimating Compositions and Nutritional Values of Seed Mixes Based on Vision Transformers
Source: Plant Phenomics. 2023 Nov 10;5:0112. doi: 10.34133/plantphenomics.0112 (PMC10637763; doi:10.34133/plantphenomics.0112)
Supplement: Supplementary 1 — Table S1 [file plantphenomics.0112.f1.docx]

The following table is provided in order to describe the different seed mixes containing different compositions of seed categories, the number of used seed categories in the mixes and the number of images containing the mixes distributed in the training and validation dataset. The table 1 demonstrates the fact that majority of the images from the same mix belongs to either the training dataset or the validation dataset. The short form of the seed names are used in the table because of the space constraints. The 12 seed categories are Tritical, Oat, Barley, Wheat, Rye, Spelt, Forage Peas, Protein Peas’, Faba Bean, Vetch, Lupine and Others.

Table 1: Different combinations of the seed categories and their distribution in the training and validation dataset

|  | |  |  |  |
| --- | --- | --- | --- | --- |
|  | Seed |  |  |  |
| Categories | Nb of |  |  |  |
| Seed Categories | Nb of Images |  |  |  |
| in Training Set | Nb of Images |  |  |  |
| in Validation Set | |  |  |  |
|  | |  |  |  |
| 0 | Tri, Oat, Bar, Rye, Spe, FP, FB, Oth | 8 | 25 | 0 |
| 1 | Tri, Oat, Whe, FP, FB, Vet, Oth | 7 | 24 | 0 |
| 2 | Tri, Oat, Bar, Spe, FP, PP, FB | 7 | 25 | 0 |
| 3 | Tri, Oat, Bar, Spe, FP, FB, Oth | 7 | 10 | 35 |
| 4 | Tri, Bar, Whe, FP, FB, Vet, Oth | 7 | 15 | 0 |
| 5 | Tri, Bar, Spe, FP, FB, Vet, Oth | 7 | 0 | 10 |
| 6 | Tri, Oat, Whe, Spe, FP, Vet | 6 | 0 | 25 |
| 7 | Tri, Oat, Whe, Spe, FP, FB | 6 | 0 | 25 |
| 8 | Tri, Oat, Whe, FP, FB, Oth | 6 | 25 | 0 |
| 9 | Tri, Oat, Spe, PP, FB, Oth | 6 | 25 | 0 |
| 10 | Tri, Oat, Spe, FP, FB, Oth | 6 | 50 | 5 |
| 11 | Tri, Oat, Rye, Spe, FP, FB | 6 | 30 | 0 |
| 12 | Tri, Oat, Rye, FP, Vet, Oth | 6 | 25 | 0 |
| 13 | Tri, Oat, FP, FB, Vet, Oth | 6 | 25 | 5 |
| 14 | Oat, Bar, Whe, PP, FB, Oth | 6 | 5 | 0 |
| 15 | Tri, Spe, FP, FB, Oth | 5 | 25 | 15 |
| 16 | Tri, Rye, FP, Vet, Oth | 5 | 25 | 0 |
| 17 | Tri, Rye, FP, FB, Oth | 5 | 25 | 0 |
| 18 | Tri, Oat, Spe, FP, FB | 5 | 35 | 0 |
| 19 | Tri, Oat, PP, FB, Vet | 5 | 0 | 25 |
| 20 | Tri, Oat, PP, FB, Oth | 5 | 25 | 0 |
| 21 | Tri, Oat, FP, Vet, Oth | 5 | 17 | 0 |
| 22 | Tri, Oat, FP, FB, Vet | 5 | 25 | 0 |
| 23 | Tri, Oat, FP, FB, Oth | 5 | 57 | 25 |
| 24 | Tri, Oat, Bar, FB, Oth | 5 | 5 | 0 |
| 25 | Tri, Bar, PP, Vet, Oth | 5 | 25 | 0 |
| 26 | Tri, Bar, PP, FB, Oth | 5 | 20 | 0 |
| 27 | Tri, Bar, FP, Vet, Oth | 5 | 25 | 0 |
| 28 | Oat, Whe, Spe, PP, FB | 5 | 25 | 0 |
| 29 | Oat, Whe, FP, Vet, Oth | 5 | 0 | 25 |
| 30 | Oat, Whe, FP, FB, Oth | 5 | 25 | 0 |
| 31 | Oat, Spe, FP, Vet, Oth | 5 | 25 | 0 |
| 32 | Oat, Bar, Whe, FB, Vet | 5 | 25 | 0 |
| 33 | Oat, Bar, FP, FB, Oth | 5 | 25 | 0 |
| 34 | Oat, Bar, FB, Vet, Oth | 5 | 25 | 0 |
| 35 | Whe, Rye, FP, Vet | 4 | 25 | 0 |
| 36 | Whe, FP, FB, Oth | 4 | 25 | 0 |
| 37 | Tri, Spe, FP, Vet | 4 | 25 | 0 |
| 38 | Tri, Spe, FP, FB | 4 | 55 | 15 |
| 39 | Tri, PP, FB, Vet | 4 | 50 | 0 |
| 40 | Tri, PP, FB, Oth | 4 | 25 | 0 |
| 41 | Tri, Oat, Spe, FP | 4 | 10 | 0 |
| 42 | Tri, Oat, PP, Vet | 4 | 0 | 27 |
| 43 | Tri, Oat, FP, Vet | 4 | 40 | 25 |
| 44 | Tri, Oat, FP, FB | 4 | 96 | 25 |
| 45 | Tri, FP, Vet, Oth | 4 | 25 | 0 |
| 46 | Tri, FP, FB, Oth | 4 | 75 | 25 |
| 47 | Tri, Bar, PP, FB | 4 | 25 | 0 |
| 48 | Oat, Whe, Spe, FP | 4 | 0 | 25 |
| 49 | Oat, PP, Vet, Oth | 4 | 25 | 0 |
| 50 | Oat, FP, Vet, Oth | 4 | 50 | 0 |
| 51 | Oat, Bar, PP, Vet | 4 | 25 | 0 |
| 52 | Oat, Bar, FP, FB | 4 | 15 | 0 |
| 53 | Bar, Rye, PP, Vet | 4 | 25 | 0 |
| 54 | Bar, FP, Vet, Oth | 4 | 25 | 0 |
| 55 | Whe, PP, Vet | 3 | 25 | 0 |
| 56 | Whe, PP, FB | 3 | 35 | 0 |
| 57 | Whe, FP, Vet | 3 | 0 | 25 |
| 58 | Whe, FP, FB | 3 | 0 | 25 |
| 59 | Whe, FB, Vet | 3 | 26 | 0 |
| 60 | Tri, Spe, FP | 3 | 0 | 25 |
| 61 | Tri, Spe, FB | 3 | 25 | 0 |
| 62 | Tri, PP, Vet | 3 | 25 | 0 |
| 63 | Tri, Oat, FP | 3 | 120 | 25 |
| 64 | Tri, Oat, FB | 3 | 25 | 0 |
| 65 | Tri, FP, Vet | 3 | 50 | 0 |
| 66 | Tri, FP, Oth | 3 | 35 | 0 |
| 67 | Tri, FB, Vet | 3 | 25 | 0 |
| 68 | Tri, Bar, FB | 3 | 25 | 0 |
| 69 | Spe, PP, Vet | 3 | 25 | 0 |
| 70 | Spe, PP, FB | 3 | 25 | 0 |
| 71 | Spe, FP, Vet | 3 | 0 | 25 |
| 72 | Spe, FP, FB | 3 | 25 | 0 |
| 73 | Spe, FB, Vet | 3 | 25 | 0 |
| 74 | Rye, PP, Vet | 3 | 0 | 25 |
| 75 | Rye, PP, FB | 3 | 25 | 0 |
| 76 | Rye, FP, Vet | 3 | 25 | 0 |
| 77 | Rye, FP, FB | 3 | 25 | 0 |
| 78 | Rye, FB, Vet | 3 | 25 | 0 |
| 79 | PP, FB, Oth | 3 | 25 | 0 |
| 80 | Oat, Spe, Vet | 3 | 25 | 0 |
| 81 | Oat, Rye, FB | 3 | 25 | 0 |
| 82 | Oat, PP, FB | 3 | 25 | 0 |
| 83 | Oat, FP, FB | 3 | 25 | 0 |
| 84 | Oat, FB, Vet | 3 | 0 | 25 |
| 85 | Bar, PP, Vet | 3 | 25 | 0 |
| 86 | Bar, FP, Vet | 3 | 0 | 25 |
| 87 | Bar, FP, FB | 3 | 25 | 0 |
| 88 | Bar, FB, Vet | 3 | 25 | 0 |
| 89 | Whe, Vet | 2 | 25 | 0 |
| 90 | Whe, Lup | 2 | 25 | 0 |
| 91 | Whe, FP | 2 | 25 | 0 |
| 92 | Whe, FB | 2 | 25 | 0 |
| 93 | Tri, Vet | 2 | 0 | 25 |
| 94 | Tri, Lup | 2 | 0 | 25 |
| 95 | Tri, FP | 2 | 40 | 25 |
| 96 | Tri, FB | 2 | 25 | 27 |
| 97 | Spe, Vet | 2 | 25 | 0 |
| 98 | Spe, Lup | 2 | 25 | 0 |
| 99 | Spe, FP | 2 | 25 | 0 |
| 100 | Spe, FB | 2 | 25 | 0 |
| 101 | Rye, Vet | 2 | 25 | 0 |
| 102 | Rye, Lup | 2 | 25 | 0 |
| 103 | Rye, FP | 2 | 24 | 0 |
| 104 | Rye, FB | 2 | 25 | 0 |
| 105 | PP, Vet | 2 | 25 | 0 |
| 106 | Oat, Vet | 2 | 25 | 0 |
| 107 | Oat, Lup | 2 | 50 | 0 |
| 108 | Oat, FP | 2 | 25 | 25 |
| 109 | Oat, FB | 2 | 25 | 25 |
| 110 | Oat, Bar | 2 | 0 | 25 |
| 111 | FP, Vet | 2 | 20 | 0 |
| 112 | FP, FB | 2 | 25 | 0 |
| 113 | FB, Vet | 2 | 25 | 0 |
| 114 | Bar, PP | 2 | 25 | 0 |
| 115 | Bar, Lup | 2 | 26 | 0 |
| 116 | Bar, FB | 2 | 25 | 0 |
| 117 | Whe | 1 | 75 | 25 |
| 118 | Vet | 1 | 95 | 5 |
| 119 | Tri | 1 | 75 | 25 |
| 120 | Spe | 1 | 75 | 25 |
| 121 | Rye | 1 | 75 | 25 |
| 122 | PP | 1 | 75 | 25 |
| 123 | Oat | 1 | 200 | 0 |
| 124 | Lup | 1 | 75 | 25 |
| 125 | FP | 1 | 100 | 25 |
| 126 | FB | 1 | 75 | 25 |
| 127 | Bar | 1 | 25 | 25 |
